# The Prescription Characteristics, Efficacy and Safety of Spironolactone in Real-World Patients With Acute Heart Failure Syndrome: A Prospective Nationwide Cohort Study

**DOI:** 10.3389/fcvm.2022.791446

**Published:** 2022-02-22

**Authors:** Soo Jin Na, Jong-Chan Youn, Hye Sun Lee, Soyoung Jeon, Hae-Young Lee, Hyun-Jai Cho, Jin-Oh Choi, Eun-Seok Jeon, Sang Eun Lee, Min-Seok Kim, Jae-Joong Kim, Kyung-Kuk Hwang, Myeong-Chan Cho, Shung Chull Chae, Seok-Min Kang, Dong-Ju Choi, Byung-Su Yoo, Kye Hun Kim, Byung-Hee Oh, Sang Hong Baek

**Affiliations:** ^1^Department of Critical Care Medicine, Samsung Medical Center, Sungkyunkwan University School of Medicine, Seoul, South Korea; ^2^Department of Medicine, Catholic University Graduate School, Seoul, South Korea; ^3^Division of Cardiology, Department of Internal Medicine, Seoul St. Mary's Hospital, Catholic Research Institute for Intractable Cardiovascular Disease, College of Medicine, The Catholic University of Korea, Seoul, South Korea; ^4^Biostatistics Collaboration Unit, Yonsei University College of Medicine, Seoul, South Korea; ^5^Department of Internal Medicine, Seoul National University Hospital, Seoul, South Korea; ^6^Department of Internal Medicine, Sungkyunkwan University College of Medicine, Seoul, South Korea; ^7^Department of Internal Medicine, Asan Medical Center, University of Ulsan College of Medicine, Seoul, South Korea; ^8^Department of Internal Medicine, Chungbuk National University College of Medicine, Cheongju, South Korea; ^9^Department of Internal Medicine, Kyungpook National University College of Medicine, Daegu, South Korea; ^10^Department of Internal Medicine, Yonsei University College of Medicine, Seoul, South Korea; ^11^Department of Internal Medicine, Seoul National University Bundang Hospital, Seongnam, South Korea; ^12^Department of Internal Medicine, Yonsei University Wonju College of Medicine, Wonju, South Korea; ^13^Department of Cardiovascular Medicine, Chonnam National University Medical School, Gwangju, South Korea; ^14^Department of Internal Medicine, Mediplex Sejong Hospital, Incheon, South Korea

**Keywords:** acute heart failure syndrome, spironolactone, mineralocorticoid receptor antagonists, drug therapy, outcome

## Abstract

**Background:**

Randomized clinical trials of spironolactone showed significant mortality reduction in patients with heart failure with reduced ejection fraction. However, its role in acute heart failure syndrome (AHFS) is largely unknown.

**Aim:**

To investigate the prescription characteristics, efficacy and safety of spironolactone in real-world patients with AHFS.

**Methods:**

5,136 AHFS patients who survived to hospital discharge using a nationwide prospective registry in Korea were analyzed. The primary efficacy outcome was 3-year all-cause mortality.

**Results:**

Spironolactone was prescribed in 2,402 (46.8%) at discharge: <25 mg in 890 patients (37.1%), ≥25 mg, and <50 mg in 1,154 patients (48.0%), and ≥50 mg in 358 patients (14.9%). Patients treated with spironolactone had a lower proportion of chronic renal failure and renal replacement therapy during hospitalization and had lower serum creatinine level than those who did not. In overall patients, 3-year mortality was not different in both groups (35.9 vs. 34.5%, *P* = 0.279). The incidence of renal injury and hyperkalemia was 2.2% and 4.3%, respectively, at the first follow-up visit. The treatment effect of spironolactone on mortality was different across subpopulations according to LVEF. The use of spironolactone was associated with a significant reduction in 3-year morality in patients with LVEF ≤ 26% (33.8 vs. 44.3%, *P* < 0.001; adjusted HR 0.79, 95% CI 0.64–0.97, *P* = 0.023), but not in patients with LVEF > 26%.

**Conclusions:**

Although spironolactone was frequently used at lower doses in real-world practice, use of spironolactone significantly reduced 3-year mortality in patients with severely reduced LVEF with acceptable safety profile. However, our findings remain prone to various biases and further prospective randomized controlled studies are needed to confirm these findings.

## Introduction

Aldosterone has gained interest as a therapeutic target due to its independent and significant role in the pathophysiology of heart failure (HF). Beyond maintaining sodium and water homeostasis, aldosterone is involved in myocardial hypertrophy, fibrosis, and endothelial dysfunction (1). After the results of the Randomized Aldactone Evaluation Study (RALES) trial, which demonstrated an association between spironolactone and considerable mortality risk reduction in patients with severe HF, mineralocorticoid antagonists became a component of treatment for HF with reduced ejection fraction (HFrEF) (2–4). There was an attempt to reconsider for spironolactone to expand its therapeutic range to HF with preserved ejection fraction (HFpEF), and recently the U.S Food and Drug Administration's advisory committee reviewed a labeled indication for spironolactone in the treatment of adults with HFpEF (5).

However, data on the efficacy and safety of spironolactone in patients with acute heart failure syndrome (AHFS) including HFpEF are still limited. Even in the Aldosterone Antagonist Therapy for Adults with Heart Failure and Preserved Systolic Function (TOPCAT) trial, which investigated the use of spironolactone in HFpEF, outcome improvement was identified only in patients enrolled in the Americas, not all participants (6, 7). In addition, spironolactone showed conflicting results in a broad unselected population with HF outside of clinical trials (8). Considering the potential risk of adverse effects of spironolactone, such as renal impairment and hyperkalemia (9, 10), it is necessary to collect data on the efficacy and safety of spironolactone in real clinical AHFS practice to establish guidance for the use of spironolactone.

Therefore, we aimed to present the current spironolactone prescription pattern, efficacy, and safety, and to evaluate whether the efficacy of spironolactone could be varied depending on the left ventricular ejection fraction (LVEF) in Korean patients with AHFS.

## Methods

### Study Design and Population

Data for this study were from the Korean Acute Heart Failure (KorAHF) registry. Details on the study design and rationale of the KorAHF registry were previously reported (11, 12). The KorAHF registry is a nationwide prospective multicenter cohort study that evaluates the clinical characteristics, management, and outcomes of patients hospitalized for AHFS in Korea. Patients were enrolled at 10 tertiary university-affiliated hospitals from March 2011 to February 2014. Patients with signs or symptoms of HF and either (1) lung congestion defined as congestion on a chest X-ray or as rales on physical examination or (2) objective findings of LV systolic dysfunction or structural heart disease were eligible for the registry. A total of 5,625 consecutive patients were enrolled in the registry. Because our study aimed to identify whether spironolactone have a homogeneous treatment effect among patient subpopulations with different ejection fraction, we included the whole range of LVEF. Among them, patients without documented data of LVEF and patients who died during index hospitalization were excluded in this study (Supplementary Figure S1). The institutional review board or ethics committee at each participating hospital approved the study protocol and waived the need for written informed consent. This study complied with the Declaration of Helsinki principles.

### Data Collection and Clinical Outcomes

Data were collected by attending physicians in each participating center using a web-based case-report form in the Clinical Research and Trial Management System (iCReaT) supported by the Korean National Institute of Health with the assistance of a clinical research coordinator. Information about patient demographic characteristics including comorbidities, etiology of HF, vital signs, laboratory and echocardiographic measurements, treatments, and clinical outcomes were obtained prospectively at the time of admission, discharge, and during the follow-up period. Data were periodically reviewed by an independent data monitoring team.

For C-reactive protein (CRP)/ high sensitivity CRP (hs-CRP) and brain natriuretic peptide (BNP)/ N-terminal pro-BNP (NT-proBNP), only one of the two variables was measured for each hospital. Therefore, these variables were classified as follows, referring to previous publications, in order to reduce missing values: BNP < 100 pg/mL or NT-proBNP < 360 pg/mL and BNP ≥ 100 pg/mL or NT-proBNP ≥ 360 pg/mL, and CRP level ≤ 10 mg/L or hsCRP ≤ 3.0 mg/L and CRP level > 10 mg/L or hsCRP > 3.0 mg/L (6, 13, 14).

The primary outcome in this study was all-cause mortality 3 years after hospital discharge. The mortality data for patients who were lost to follow-up was collected from the National Insurance data or National Death Records. Mortality and cause of death were verified by a Clinical Event Committee, which was composed of independent experts in HF who have not participated in patient enrolment.

### Definitions

The use of spironolactone was assessed at hospital discharge. The prescription for initiation, dose adjustment or discontinuation of medications including spironolactone was left to the discretion of the physician in charge, but the decision-making generally followed the guidelines (3, 4). The safety of spironolactone treatment was evaluated at the first post-discharge outpatient follow-up visit. Renal injury was defined as a doubling of serum creatinine based on the Risk, Injury, Failure, Loss, and End-stage Kidney (RIFLE) classification creatinine doubling, and hyperkalemia was defined as potassium greater than 5.5 mmol/L (15).

Echocardiography was performed by a board-certified cardiologist or echocardiography technician. Quantitative assessment of LVEF using the modified Simpson's method was recommended, but LVEF that measured by M-mode or visual estimation was also used for HF categorization when the accuracy of the biplane method was limited due to a poor acoustic window (16). We defined the HF classification based on LVEF using the criteria of American College of Cardiology/American Heart Association (ACC/AHA) and the European Society of Cardiology (ESC) guidelines as follows: HFrEF, HF with LVEF ≤40%; HF with mid-range ejection fraction (HFmrEF), HF with LVEF 41–49%, HFpEF, HF with LVEF ≥50% (4, 17).

### Statistical Analysis

To compare clinical characteristics and outcomes between the two groups, we analyzed categorical variables as numbers and percentages using the χ^2^ test or Fisher's exact test. Continuous variables are reported as mean ± standard deviation and were compared using the *t*-test. We performed subpopulation treatment effect pattern plot (STEPP) and the Contal and O'Quigley to evaluate whether the effect of spironolactone varies according to LVEF and to identify the cut-off value. To evaluate the effect of the spironolactone use and to identify risk factors for the 3-year all-cause mortality, we performed Cox proportional hazard regression analysis. Variables deemed clinically relevant from previous studies were considered candidate variables in multivariable Cox regression models. The hazard ratio (HR) of each variable is reported with the 95% confidence interval (CI). Survival curves were constructed by the Kaplan-Meier method, and the significance level was assessed using the log rank test to assess the effect of spironolactone with respect to the primary outcome according to classification of HF. To reduce the effects of potential confounders and selection bias, we performed a sensitivity analysis using propensity score matching. Propensity scores were estimated using a logistic regression model of the treatment on the covariate included in the Cox regression models. The patients were matched 1:1 by propensity scores. For all analyses, a two-tailed test with a *P*-value less than 0.05 was considered statistically significant. Statistical analyses were performed using SAS version 9.4 (SAS Institute, Cary, NC, USA) and R software package (R Foundation for Statistical Computing, Vienna, Austria).

## Results

### Prescription Pattern of Spironolactone

Of the 5,136 eligible patients, 2,402 (46.8%) patients were treated with spironolactone. The proportion of patients prescribed spironolactone decreased as the degree of renal function worsened (Supplementary Figure S2). The prescribed doses of spironolactone were < 25 mg in 890 (37.1%), ≥ 25 mg and < 50 mg in 1,154 (48.0%), and ≥ 50 mg in 358 (14.9%) in overall patients. About 75% of survivors in the spironolactone group were followed up until 3 years after hospital discharge, and 51.8% of them maintained spironolactone treatment during the 3-year follow-up period (Supplementary Figure S3).

There were significant differences in clinical and in-hospital treatment characteristics between patients treated with spironolactone and without spironolactone (Table 1). The proportion of de novo HF, hypertension, diabetes, and ischemic heart disease were higher in the no spironolactone group, and the proportion of dilated cardiomyopathy, and atrial fibrillation were higher in the spironolactone group. In the spironolactone group, chronic renal failure and need of renal replacement therapy during hospitalization were less common and serum creatinine was significantly lower than in the no spironolactone group. In addition, the spironolactone group had lower LVEF than the no spironolactone group.

**Table 1:** Clinical and treatment characteristics in overall patients.

**Variables**	**Overall**	**No SPR**	**SPR**	***P-*Value**
	**(*n* = 5,136)**	**(*n* = 2,734)**	**(*n* = 2,402)**	
Age, years	68.4 ± 14.4	69.0 ± 14.4	67.7 ± 14.4	<0.001
Male	2728 (53.1)	1463 (53.5)	1265 (52.7)	0.544
De novo HF	2748 (53.5)	1509 (55.2)	1239 (51.6)	0.010
**Past medical history**				
Hypertension	3025 (58.9)	1682 (61.5)	1343 (55.9)	<0.001
Diabetes mellitus	1799 (35.0)	1015 (37.1)	784 (32.6)	0.001
Ischemic heart disease	1415 (27.6)	793 (29.0)	622 (25.9)	0.014
Dilated cardiomyopathy	411 (8.0)	172 (6.3)	239 (10.0)	<0.001
Valvular heart disease	724 (14.1)	390 (14.3)	334 (13.9)	0.716
Atrial fibrillation	1456 (28.4)	739 (27.0)	717 (29.9)	0.025
Chronic lung disease	567 (11.0)	314 (11.5)	253 (10.5)	0.280
Chronic renal failure	698 (13.6)	515 (18.8)	183 (7.6)	<0.001
Cerebrovascular disease	764 (14.9)	428 (15.7)	336 (14.0)	0.093
**Treatment during hospitalization**				
Parenteral diuretics	3843 (74.8)	1979 (72.4)	1864 (77.6)	<0.001
Parenteral inotropes	1463 (28.5)	764 (27.9)	699 (29.1)	0.360
Parenteral vasodilators	2124 (41.4)	1232 (45.1)	892 (37.1)	<0.001
Intensive care unit admission	2412 (47.0)	1345 (49.2)	1067 (44.4)	0.001
Mechanical ventilation	670 (13.1)	387 (14.2)	283 (11.8)	0.012
Renal replacement therapy	286 (5.6)	231 (8.5)	55 (2.3)	<0.001
**Vital signs at discharge**				
Systolic blood pressure, mmHg	114.9 ± 17.6	117.3 ± 17.7	112.2 ± 17.1	<0.001
Heart rate, /min	76.8 ± 14.1	77.5 ± 14.3	76.0 ± 13.9	0.062
NYHA class II-IV	4224 (82.2)	2242 (82.0)	1982 (82.5)	0.633
**Laboratory measurements at discharge**				
Sodium, mmol/L	137.9 ± 3.9	138.1 ± 3.9	137.7 ± 4.0	0.161
Potassium, mmol/L	4.2 ± 0.5	4.2 ± 0.5	4.2 ± 0.5	0.154
Hemoglobin, g/dL	12.1 ± 2.1	11.9 ± 2.1	12.4 ± 2.1	<0.001
Creatinine, mg/dL	1.35 ± 1.31	1.58 ± 1.66	1.09 ± 0.64	<0.001
CRP > 3mg/dL or hs-CRP > 10 mg/dL	555 (11.4)	332 (12.6)	223 (10.0)	0.004
BNP > 100pg/mL or NT-proBNP > 360 pg/mL	4425 (94.9)	2328 (93.7)	2097 (96.2)	<0.001
**Echocardiographic parameters**				
LVEDV, mL	151.8 ± 71.8	142.0 ± 65.0	164.9 ± 78.1	<0.001
LVESV, mL	99.2 ± 62.6	91.1 ± 57.4	110.1 ± 67.5	<0.001
Ejection fraction, %	37.9 ± 15.5	39.9 ± 15.5	35.7 ± 15.1	<0.001
LA volume index, mL/m^2^	63.7 ± 42.2	61.2 ± 33.9	66.1 ± 48.8	0.002
E, m/sec	0.94 ± 0.39	0.94 ± 0.40	0.95 ± 0.37	0.250
A, m/sec	0.76 ± 1.51	0.76 ± 0.37	0.76 ± 2.24	0.981
E/A ratio	1.6 ± 3.8	1.5 ± 2.9	1.8 ± 4.6	0.018
Deceleration time, msec	170.7 ± 82.9	176.3 ± 88.2	164.3 ± 76.0	<0.001
e', cm/sec	5.01 ± 2.32	5.05 ± 2.13	4.96 ± 2.52	0.249
a', cm/sec	6.16 ± 2.76	6.44 ± 2.70	5.79 ± 2.79	<0.001
E/e' ratio	21.2 ± 11.4	20.9 ± 11.4	21.5 ± 11.5	0.070
TR Vmax, m/s	2.90 ± 0.59	2.88 ± 0.57	2.91 ± 0.61	0.202

*Values are mean ± standard deviation and median with interquartile range or n (%)*.

*BNP, brain natriuretic peptide; CRP, C-reactive protein; HF, heart failure; hs-CRP, high-sensitivity C-reactive protein; NT-proBNP, N-terminal pro-brain natriuretic peptide; NYHA, New York Heart Association; SPR, spironolactone*.

### Mortality and Adverse Events

**Figure 1:**
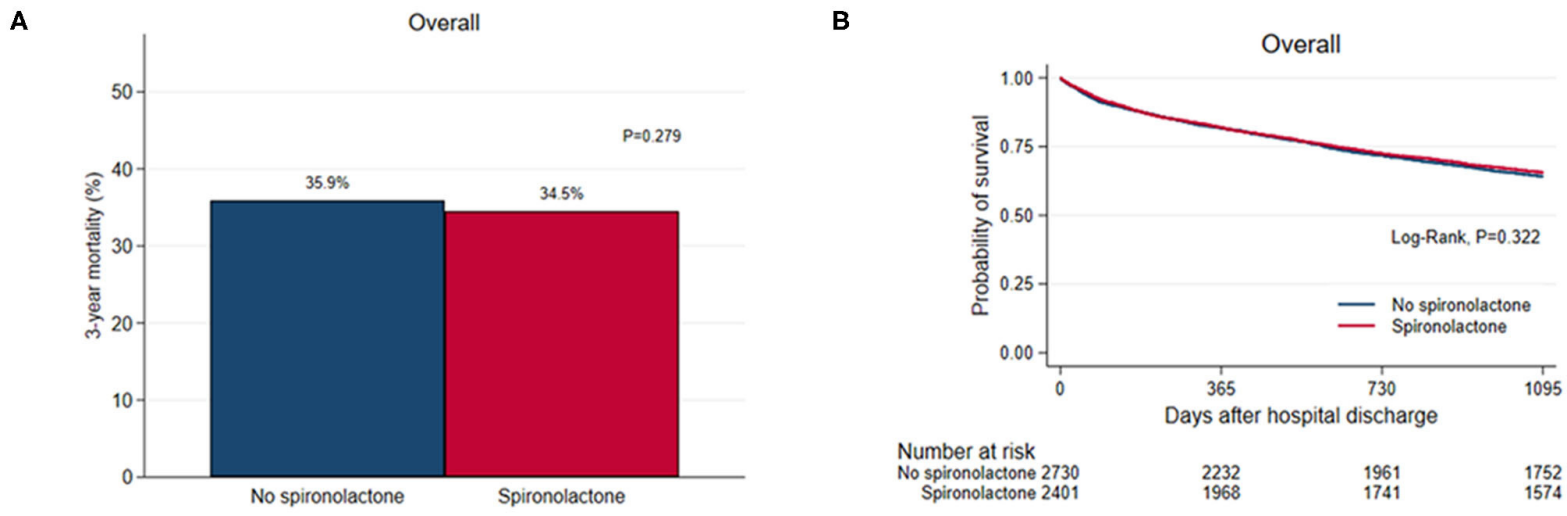
**(A)** Bar graph and **(B)** Kaplan–Meier curves of the 3-year all-cause mortality after hospital discharge according to spironolactone treatment in overall patients.

Overall, 1,810 (35.2%) patients died during the 3-year follow-up after discharge and there was no significant difference in 3-year (35.9% vs. 34.5%, *P* = 0.279) all-cause mortality between the two groups (Figure 1). As renal function worsened, the 3-year mortality tended to increase and the difference in 3-year mortality between the two groups was not significant except for patients with glomerular filtration rate <15 mL/min/1.73m^2^ (57/132 vs. 2/14, *P* = 0.036) (Supplementary Table S1).

Among the spironolactone group, 1,324 (55.1%) patients had post-discharge outpatient follow-up visits with blood sampling within an average of 14.9 days. There were significant differences in systolic blood pressure (113.1 mmHg vs. 109.9 mmHg, *P* < 0.001), serum creatinine (1.08 mg/dL vs. 1.18 mg/dL, *P* < 0.001), and serum potassium (4.2 mmol/L vs. 4.6 mmol/L, *P* < 0.001) compared with those at hospital discharge, but the incidence of renal injury and hyperkalemia was 2.2 and 4.3%, respectively (Supplementary Table S2). There was no case of discontinuing spironolactone due to gynecomastia in the transition period (Supplementary Table S3).

### Effect of Spironolactone According to LVEF

**Figure 2:**
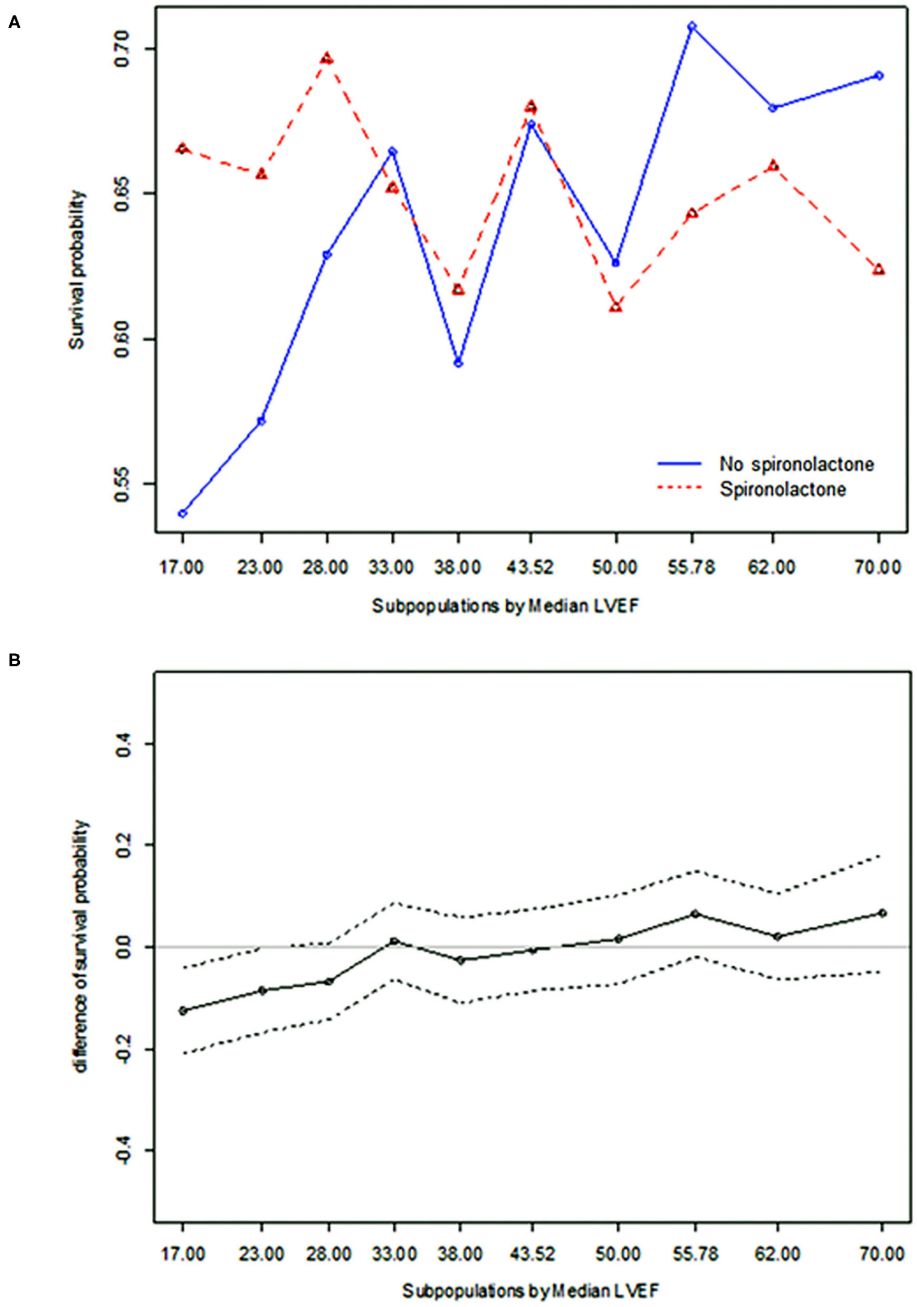
Subpopulation treatment effect pattern plot analysis of the treatment effect of spironolactone as measured by **(A)** 3-year all-cause mortality, **(B)** difference in 3-year all-cause mortality.

**Figure 3:**
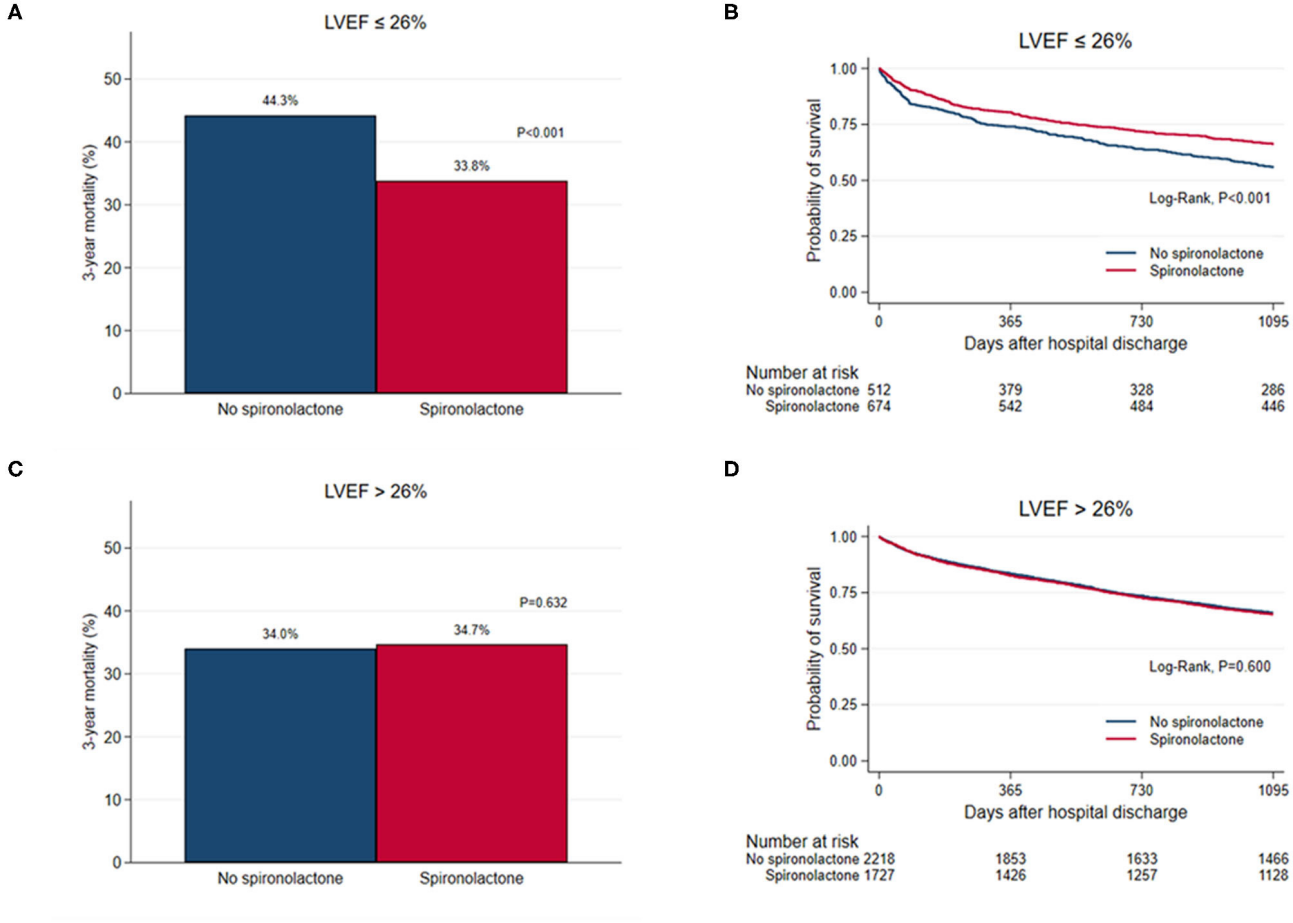
**(A,C)** Bar graph and **(B,D)** Kaplan–Meier curves of the 3-year all-cause mortality after hospital discharge according to spironolactone treatment in patients with left ventricular ejection fraction (LVEF) ≤ 26% and in patients with LVEF > 26%.

STEPP analysis of the treatment effect of spironolactone across subpopulations according to LVEF showed that the spironolactone treatment was associated with lower 3-year mortality only in subpopulations where LVEF was less than 28% (Figure 2 and Supplementary Table S4). The most significant cut-off value of LVEF by Contal and O'Quigley method was 26.1%, which discriminated patients with different survival according to use of spironolactone. In patients with LVEF < 26.1%, the spironolactone group had significantly lower 3-year (33.8 vs. 44.3%, *P* < 0.001) mortality than the no spironolactone group, but there was no difference in mortality between the two groups in patients with LVEF > 26% (Figure 3). Cox regression analysis revealed that spironolactone treatment was independently associated with a reduction in 3-year mortality in patients with LVEF ≤ 26% (HR 0.71, adjusted HR 0.79, 95% CI 0.64–0.97, *P* = 0.023) (Table 2).

**Table 2:** Predictors for 3-year all-cause mortality in patients according to LVEF.

	**In patients with LVEF** **≤26%**					
	**Univariable**			Multivariable^*^		
**Variables**	**HR**	**95% CI**	***P*-value**	**Adjusted HR**	**95% CI**	***P*-value**
Spironolactone use	0.71	0.59–0.86	<0.001	0.79	0.64–0.97	0.023
Age ≥67	3.20	2.59–3.94	<0.001	2.78	2.16–3.56	<0.001
Male	0.89	0.73–1.08	0.232	0.86	0.69–1.06	0.157
De novo HF	0.44	0.36–0.54	<0.001	0.66	0.53–0.82	<0.001
Hypertension	1.73	1.43–2.09	<0.001	1.33	1.07–1.67	0.012
Diabetes mellitus	1.62	1.35–1.95	<0.001	1.35	1.09–1.66	0.005
Cerebrovascular disease	1.75	1.39–2.21	<0.001	1.18	0.91–1.53	0.209
Use of parenteral inotropes	1.75	1.46–2.10	<0.001	1.32	1.06–1.63	0.011
Systolic blood pressure	1.00	0.99–1.00	0.353	0.99	0.98–1.00	0.015
Heart rate	1.01	1.00–1.02	0.001	1.01	1.01–1.02	0.001
Sodium	0.92	0.91–0.94	<0.001	0.95	0.93–0.97	<0.001
CRP > 3 mg/dL or hs-CRP > 10 mg/dL	1.96	1.48–2.60	<0.001	1.51	1.12–2.04	0.007
BNP > 100 pg/mL or NT-proBNP > 360 pg/mL	20.43	1.28–326.50	0.033	13.55	0.84–218.77	0.066
	**In patients with LVEF** **>** **26%**					
	**Univariable**			Multivariable^*^		
**Variables**	**HR**	**95% CI**	***P*** **-value**	**Adjusted HR**	**95% CI**	***P*** **-value**
Spironolactone use	1.03	0.92–1.14	0.653	1.00	0.89–1.12	0.995
Age ≥67	2.36	2.11–2.64	<0.001	2.13	1.88–2.42	<0.001
Male	0.99	0.89–1.10	0.870	1.09	0.97–1.23	0.142
De novo HF	0.61	0.55–0.68	<0.001	0.66	0.59–0.74	<0.001
Hypertension	1.41	1.26–1.59	<0.001	1.09	0.95–1.24	0.216
Diabetes mellitus	1.33	1.19–1.48	<0.001	1.12	0.99–1.26	0.065
Cerebrovascular disease	1.58	1.38–1.80	<0.001	1.35	1.18–1.56	<0.001
Use of parenteral inotropes	1.02	0.90–1.15	0.739	0.99	0.87–1.13	0.895
Systolic blood pressure	1.00	1.00–1.00	0.118	1.00	1.00–1.00	0.730
Heart rate	1.01	1.00–1.01	<0.001	1.01	1.00–1.01	<0.001
Sodium	0.93	0.92–0.95	<0.001	0.95	0.93–0.96	<0.001
CRP > 3 mg/dL or hs-CRP > 10 mg/dL	1.47	1.26–1.72	<0.001	1.36	1.15–1.60	<0.001
BNP > 100 pg/mL or NT-proBNP > 360 pg/mL	2.56	1.83–3.58	<0.001	2.00	1.42–2.84	<0.001

* *All variables shown in table were entered into the multivariable Cox proportional hazard regression model*.

*BNP, brain natriuretic peptide; CI, confidence interval; CRP, C-reactive protein; HF, heart failure; HR, hazard ratio; hs-CRP, high-sensitivity C-reactive protein; LVEF, left ventricular ejection fraction; NT-proBNP, N-terminal pro-brain natriuretic peptide*.

**Figure 4:**
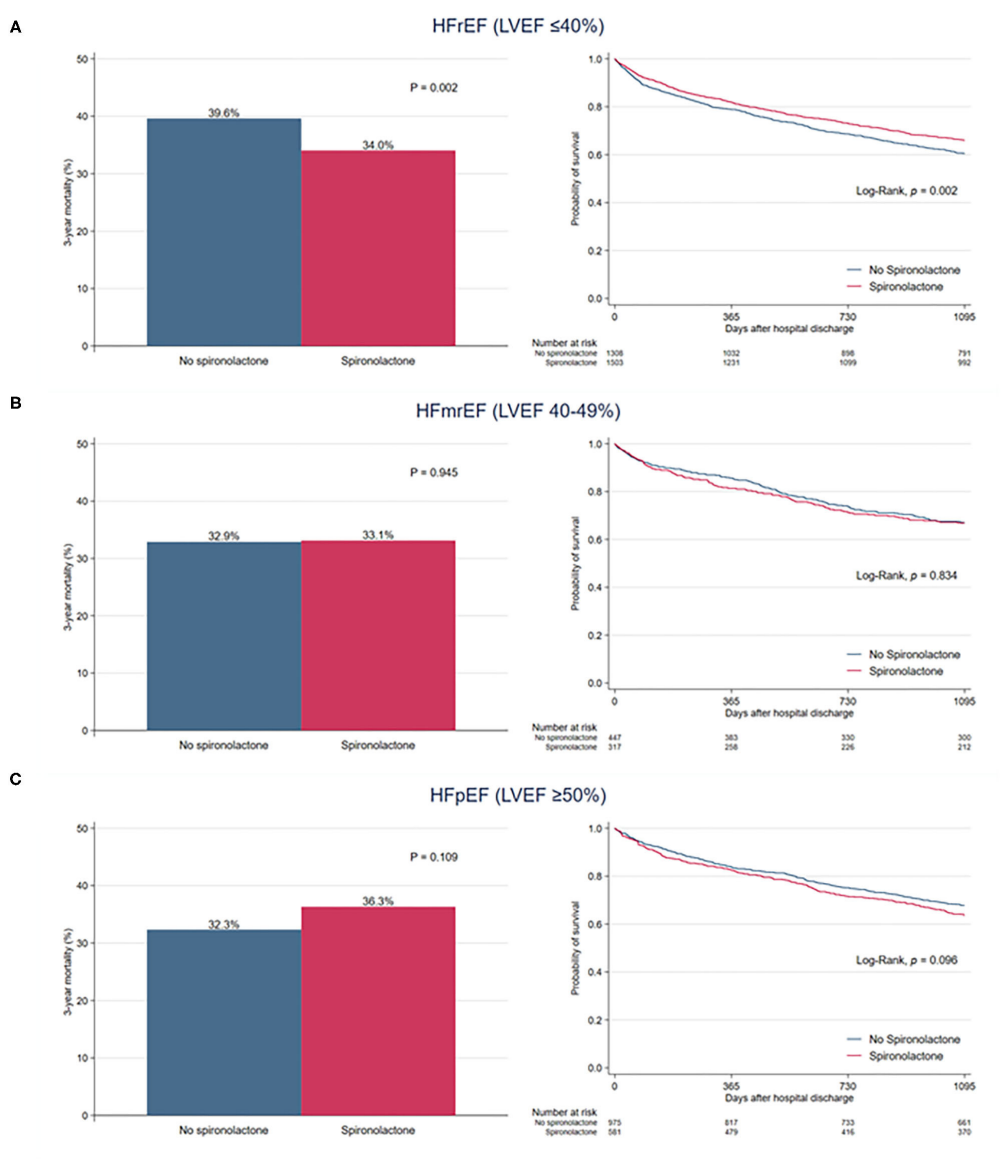
Bar graph and Kaplan–Meier curves of the 3-year all-cause mortality after hospital discharge according to spironolactone treatment in patients with **(A)** heart failure with reduced ejection fraction (HFrEF), **(B)** heart failure with mid-range ejection fraction (HFmrEF), and **(C)** heart failure with preserved ejection fraction (HFpEF).

When overall patients were divided into three classifications of HF based on LVEF, there was no significant difference in 3-year mortality between the two groups among patients with HFmrEF (32.9 vs. 33.1%, *P* = 0.945) and HFpEF (32.3 vs. 36.3%, *P* = 0.109) (Figure 4). In patients with HFrEF, the 3-year mortality rate was significantly reduced in the spironolactone group (34.0 vs. 39.6%, *P* = 0.002).

### Sensitivity Analysis

Clinical and in-hospital treatment characteristics of the two groups in propensity score matched cohort are shown in the Supplementary Table S5. The no spironolactone group had a higher proportion of chronic renal failure and renal replacement therapy during hospitalization than the spironolactone group. Potassium and creatinine levels and LVEF at discharge were also slightly higher in the no spironolactone group. Similar to the results of our main analysis, the Kaplan-Meier survival curve showed a significant difference between the survival rates of the two groups during a 3-year follow up in patients with LVEF ≤ 26% (log rank test, *P* = 0.043), but the survival rates were similar between the two groups in patients with LVEF > 26% (log rank test, *P* = 0.824) (Supplementary Figure S4). In Cox regression analysis, spironolactone was related to 3-year mortality in patients with LVEF ≤ 26% (adjusted HR 0.78, 95% CI 0.62–0.99, *P* = 0.044), but not in patients with LVEF > 26% (adjusted HR 1.01, 95% CI 0.89–1.15, *P* = 0.824).

## Discussion

In the present study, we identified the prescription pattern of spironolactone and evaluated the efficacy and safety of spironolactone in patients with AFHS using a large prospective nationwide cohort in Korea. We found that (1) spironolactone was used in 46.8% of Korean patients with AHFS and spironolactone was often used at a dose lower than the dose recommended in the guidelines; (2) use of spironolactone was not associated with reduction of 3-year all-cause mortality and occurrence of significant renal injury or hyperkalemia in overall patients; and (3) the effect of spironolactone on mortality was different depending on the LVEF, and the survival benefit was particularly remarkable in patients with severely reduced LVEF.

Mineralocorticoid antagonists, including spironolactone, are commonly prescribed in HFrEF patients, which ranges from about 50 to 70% in recent studies (18–21). Spironolactone has emerged as an important treatment option for HF since the 1990s because of its ability to attenuate the neurohormonal signals, which play a central role in the progression of HF, and to reverse remodeling. Spironolactone treatment significantly reduces plasma procollagen type III aminoterminal peptide (PIIINP), a biochemical marker of myocardial fibrosis and/or remodeling, and BNP, a prognostic marker of HF, and improves endothelial function, which is associated with cardiovascular events in patients with HF of varying severity (22–24). Studies evaluating the effect of spironolactone with echocardiographic assessment showed improvement of LV systolic and diastolic function and ventricular-arterial coupling, as well as reduction of LV volume and mass in patients treated with spironolactone (25, 26). In addition to the improvement of these laboratory and echocardiographic parameters, positive results from three large-scale, multi-center, placebo-controlled clinical trials, RALES, the Eplerenone Post–Acute Myocardial Infarction Heart Failure Efficacy and Survival Study (EPHESUS), and the Eplerenone in Mild Patients Hospitalization and Survival Study in Heart Failure (EMPHASIS-HF), empowered MRA treatment in HFrEF (2, 27, 28). Furthermore, the TOPCAT trial suggested that the effect of spironolactone are not limited to HFrEF, but may extend to patients with HFpEF (6).

Contrary to the favorable results of the previous studies, spironolactone was not associated with a reduced mortality in our entire cohort. Our findings are consistent with a study of Lund et al. who failed to show differences in mortality according to mineralocorticoid antagonist treatment in a large general HF population of the Swedish Heart Failure Registry (8). It suggests that there may be a gap between the randomized clinical trials and real-world practice. In our study, patients who received spironolactone treatment had substantially different characteristics from those who did not, suggesting that spironolactone was selectively prescribed. Considering the *post-hoc* analysis of the TOPCAT trial, which demonstrated the the response to spironolactone was significantly different according to clinical phenogroups (6, 29), differences in patient selection and patient characteristics may be one explanation. In particular, we did not limit our analysis to patients with HFrEF or HFpEF because our study was interested in evaluating the efficacy of spironolactone in real-world practice, but the efficacy of spironolactone was different depending on the LVEF. The survival benefit of spironolactone was significant only in patient with severely reduced LVEF. Although our findings cannot explain the underlying mechanism of the relationship between spironolactone and LVEF, it supports the current guidelines recommending the use of spironolactone in patients with LVEF ≤ 35% (17, 30).

In our study, spironolactone was prescribed in only about half of overall patients. In particular, the proportion of patients prescribed spironolactone was significantly reduced in patients with severe renal dysfunction. Our findings are similar to a recent study by Patel et al. which demonstrated that spironolactone was infrequently used compared to other guideline recommended, especially in patients with renal dysfunction (31). It is presumably the result of concerns about spironolactone-related complications, such as worsening of renal function and hyperkalemia. However, selective use of spironolactone did not increase the incidence of mortality or adverse events in patients with renal dysfunction in our cohort. Furthermore, Oh et al. showed the survival benefit of spironolactone in patients with stage 3b chronic kidney disease (32). Therefore, further studies should be conducted for the proper use of spironolactone in patients with renal dysfunction.

Also, the that about 40% of patients treated with spironolactone in our study were not prescribed a guideline-recommended dose may have may have influenced the efficacy of spironolactone treatment. Although current guidelines recommend 25 mg spironolactone once daily with titration up to 50 mg once daily for patients with HFrEF based on landmark trials (17, 30), a large number of patients are treated with spironolactone doses of less than 25 mg in real-world practice (19, 33). The dose response relationship between spironolactone and survival has not yet been clearly identified. In the Aldosterone Targeted Neurohormonal Combined with Natriuresis Therapy in Heart Failure (ATHENA-HF) trial, 100 mg of spironolactone was not associated with an improved outcome compared to placebo or 25 mg of spironolactone in patients with AHFS (34). On the other hand, in the ASIAN-HF registry, patients who received at least 100% of guideline-recommended dose had better composite outcomes of all-cause deaths or hospitalization for HF than did those who received lower doses (19). To properly assess the characteristics of patients who are likely to benefit from spironolactone treatment, the effects of under-dosing should be further elucidated.

Although our study provided information regarding the association between spironolactone treatment and long-term survival in a large population of Korean patients with AHFS, there are several limitations that should be considered. First, because of the observational study design, our findings remain prone to various biases and potential confounding factors. Although we used regression modeling and propensity score matching to control for confounders, unmeasured confounders may have been present. In particular, our study did not control the effect of standard medical treatment for HF with reduced EF such as renin-angiotensin system blockade or beta blocker. Second, it is possible that more severe and complex patients were included in this study because only tertiary university-affiliated hospitals participated in the registry. In addition, since risk factors for mortality and the efficacy of medication in patients with HF may vary depending on regional differences, our findings have limitations in their generalizability to other populations (7). Third, we determined whether the patient was treated with spironolactone only by prescription at hospital discharge. Because the total treatment duration and changes in dosage of spironolactone and medication adherence were not evaluated, we could not exclude the possibility that insufficient treatment duration and adherence may have affected our findings. In addition, we could not assess the effect of valvular and right ventricular function on clinical outcomes. Further prospective randomized controlled studies are needed to confirm these findings.

## Conclusion

Spironolactone was prescribed in selective patients and under-dosing was common for treatment of AHFS in real-world clinical practice. Although spironolactone was used in patients with a wide range of LVEF, the effect of spironolactone on mortality differed according to the LVEF and spironolactone was associated with a reduction of 3-year mortality only in patients with severely reduced LVEF. Further studies to identify patients who are likely to benefit from spironolactone treatment are necessary for the expansion of the therapeutic field of spironolactone and the optimal use in patient with AHFS.

## Data Availability Statement

The raw data supporting the conclusions of this article will be made available by the authors, without undue reservation.

## Ethics Statement

The studies involving human participants were reviewed and approved by Institutional Review Board of the Catholic Medical Center. Written informed consent for participation was not required for this study in accordance with the national legislation and the institutional requirements.

## Author Contributions

SJN, J-CY, and SHB conceived and designed the study and drafted the manuscript for intellectual content. SJN, J-CY, HSL, SJ, and SHB analyzed and interpreted the data. SJN, J-CY, H-YL, H-JC, J-OC, E-SJ, SEL, M-SK, J-JK, K-KH, M-CC, SCC, S-MK, D-JC, B-SY, KHK, B-HO, and SHB revised the manuscript. All authors read and approved the final manuscript.

## Funding

This research was supported by the National Research Foundation of Korea (NRF) grant funded by the Ministry of Science and ICT (NRF-2021R1F1A1063430), by the Catholic Medical Center Research Foundation (2021) and by Research of Korea Centers for Disease Control and Prevention (2010-E63003-00, 2011-E63002-00, 2012-E63005-00, 2013-E63003-00, 2014-E63003-01, 2015-E63003-02, 2016-ER6303-00, and 2017-ER6303-01). The funders had no role in the study design, data collection and analysis, decision to publish, or preparation of the manuscript.

## Conflict of Interest

The authors declare that the research was conducted in the absence of any commercial or financial relationships that could be construed as a potential conflict of interest.

## Publisher's Note

All claims expressed in this article are solely those of the authors and do not necessarily represent those of their affiliated organizations, or those of the publisher, the editors and the reviewers. Any product that may be evaluated in this article, or claim that may be made by its manufacturer, is not guaranteed or endorsed by the publisher.
