# Corrigendum: The Prescription Characteristics, Efficacy and Safety of Spironolactone in Real-World Patients With Acute Heart Failure Syndrome: A Prospective Nationwide Cohort Study

**DOI:** 10.3389/fcvm.2022.888829

**Published:** 2022-04-04

**Authors:** Soo Jin Na, Jong-Chan Youn, Hye Sun Lee, Soyoung Jeon, Hae-Young Lee, Hyun-Jai Cho, Jin-Oh Choi, Eun-Seok Jeon, Sang Eun Lee, Min-Seok Kim, Jae-Joong Kim, Kyung-Kuk Hwang, Myeong-Chan Cho, Shung Chull Chae, Seok-Min Kang, Dong-Ju Choi, Byung-Su Yoo, Kye Hun Kim, Byung-Hee Oh, Sang Hong Baek

**Affiliations:** ^1^Department of Critical Care Medicine, Samsung Medical Center, Sungkyunkwan University School of Medicine, Seoul, South Korea; ^2^Department of Medicine, Catholic University Graduate School, Seoul, South Korea; ^3^Division of Cardiology, Department of Internal Medicine, Seoul St. Mary's Hospital, Catholic Research Institute for Intractable Cardiovascular Disease, College of Medicine, The Catholic University of Korea, Seoul, South Korea; ^4^Biostatistics Collaboration Unit, Yonsei University College of Medicine, Seoul, South Korea; ^5^Department of Internal Medicine, Seoul National University Hospital, Seoul, South Korea; ^6^Department of Internal Medicine, Sungkyunkwan University College of Medicine, Seoul, South Korea; ^7^Department of Internal Medicine, Asan Medical Center, University of Ulsan College of Medicine, Seoul, South Korea; ^8^Department of Internal Medicine, Chungbuk National University College of Medicine, Cheongju, South Korea; ^9^Department of Internal Medicine, Kyungpook National University College of Medicine, Daegu, South Korea; ^10^Department of Internal Medicine, Yonsei University College of Medicine, Seoul, South Korea; ^11^Department of Internal Medicine, Seoul National University Bundang Hospital, Seongnam, South Korea; ^12^Department of Internal Medicine, Yonsei University Wonju College of Medicine, Wonju, South Korea; ^13^Department of Cardiovascular Medicine, Chonnam National University Medical School, Gwangju, South Korea; ^14^Department of Internal Medicine, Mediplex Sejong Hospital, Incheon, South Korea

**Keywords:** acute heart failure syndrome, spironolactone, mineralocorticoid receptor antagonists, drug therapy, outcome

In the original article, there was an error. An author name was incorrectly spelled as “Kye Hoon Kim.” The correct spelling is “Kye Hun Kim.”

The authors apologize for this error and state that this does not change the scientific conclusions of the article in any way. The original article has been updated.

## Publisher's Note

All claims expressed in this article are solely those of the authors and do not necessarily represent those of their affiliated organizations, or those of the publisher, the editors and the reviewers. Any product that may be evaluated in this article, or claim that may be made by its manufacturer, is not guaranteed or endorsed by the publisher.

