# Supplementary material for: The Prescription Characteristics, Efficacy and Safety of Spironolactone in Real-World Patients With Acute Heart Failure Syndrome: A Prospective Nationwide Cohort Study
Source: Front Cardiovasc Med. 2022 Feb 22;9:791446. doi: 10.3389/fcvm.2022.791446 (PMC8902170; doi:10.3389/fcvm.2022.791446)
Supplement: Supplementary file 1 [file Table_1.DOCX]

**Supplementary material**

**The prescription characteristics, efficacy and safety of spironolactone in real-world patients with acute heart failure syndrome: A prospective nationwide cohort study**

Soo Jin Na, Jong-Chan Youn, Hye Sun Lee, Soyoung Jeon, Hae-Young Lee, Hyun-Jai Cho, Jin-Oh Choi, Eun-Seok Jeon, Sang Eun Lee, Min-Seok Kim, Jae-Joong Kim, Kyung-Kuk Hwang, Myeong-Chan Cho, Shung Chull Chae, Seok-Min Kang, Dong-Ju Choi, Byung-Su Yoo, Kye Hoon Kim, Byung-Hee Oh, Sang Hong Baek

**Table S1. Mortality and adverse events at the first follow-up visit according to glomerular filtration rate**

|  | **GFR ≥90mL/min/m^2^** | | | **GFR 60-89 mL/min/m^2^** | | | **GFR 30-59 mL/min/m^2^** | | |
| --- | --- | --- | --- | --- | --- | --- | --- | --- | --- |
| Variables | No SPR | SPR | P-value | No SPR | SPR | P-value | No SPR | SPR | P-value |
| 3-year mortality | 48 (15.7) | 70 (21.5) | 0.062 | 128 (26.2) | 175 (27.1) | 0.754 | 214 (41.6) | 187 (42.9) | 0.696 |
| Changes in Cr | 0.10±0.12 | 0.11±0.15 | 0.219 | 0.08±0.28 | 0.10±0.21 | 0.098 | 0.08±0.53 | 0.08±0.26 | 0.951 |
| Changes in K | 0.14±0.48 | 0.35±0.60 | <0.001 | 0.16±0.54 | 0.38±0.64 | <0.001 | 0.21±0.60 | 0.35±0.58 | <0.001 |
| Changes in SBP | 1.06±15.91 | -1.83±17.17 | 0.042 | 0.81±19.21 | -3.59±18.85 | <0.001 | 1.50±20.92 | -3.81±17.97 | <0.001 |

|  | **GFR 15-29 mL/min/m^2^** | | | **GFR < 15 mL/min/m^2^** | | |
| --- | --- | --- | --- | --- | --- | --- |
| Variables | No SPR | SPR | P-value | No SPR | SPR | P-value |
| 3-year mortality | 85 (53.5) | 37 (59.7) | 0.404 | 57 (43.2) | 2 (14.3) | 0.036 |
| Delta Cr | 0.004±0.69 | 0.17±0.71 | 0.108 | -0.11±1.60 | -0.31±1.28 | 0.663 |
| Delta K | 0.41±0.73 | 0.55±0.76 | 0.202 | 0.47±0.80 | 0.64±0.86 | 0.441 |
| Delta SBP | 0.09±20.73 | -1.54±20.32 | 0.624 | -1.91±21.88 | -4.92±15.52 | 0.630 |

Values are mean ± standard deviation or n (%).

Cr, creatinine; GFR, glomerular filtration rate; K, potassium; SBP, systolic blood pressure; SPR, spironolactone.
