# Supplementary material for: The Prescription Characteristics, Efficacy and Safety of Spironolactone in Real-World Patients With Acute Heart Failure Syndrome: A Prospective Nationwide Cohort Study
Source: Front Cardiovasc Med. 2022 Feb 22;9:791446. doi: 10.3389/fcvm.2022.791446 (PMC8902170; doi:10.3389/fcvm.2022.791446)
Supplement: Supplementary file 2 [file Table_2.DOCX]

**Supplementary material**

**The prescription characteristics, efficacy and safety of spironolactone in real-world patients with acute heart failure syndrome: A prospective nationwide cohort study**

Soo Jin Na, Jong-Chan Youn, Hye Sun Lee, Soyoung Jeon, Hae-Young Lee, Hyun-Jai Cho, Jin-Oh Choi, Eun-Seok Jeon, Sang Eun Lee, Min-Seok Kim, Jae-Joong Kim, Kyung-Kuk Hwang, Myeong-Chan Cho, Shung Chull Chae, Seok-Min Kang, Dong-Ju Choi, Byung-Su Yoo, Kye Hoon Kim, Byung-Hee Oh, Sang Hong Baek

**Table S2. Adverse events at the first follow-up visit**

| Variables | At hospital discharge | At the first follow-up | *P*-value |
| --- | --- | --- | --- |
| Systolic blood pressure, mmHg | 113.1±17.0 | 109.9±18.6 | <0.001 |
| Serum creatinine, mg/dL | 1.08±0.64 | 1.18±0.62 | <0.001 |
| Renal injury^a^ | – | 29 (2.2%) | – |
| Serum potassium, mmol/L | 4.2±0.5 | 4.6±0.6 | <0.001 |
| Hyperkalemia^b^ | – | 57 (4.3%) | – |

Values are mean ± standard deviation or n (%).

a. Renal injury was defined as a two-fold increase in serum creatinine.

b. Hyperkalemia was defined as potassium > 5.5 mmol/L.
