# Supplementary material for: The Prescription Characteristics, Efficacy and Safety of Spironolactone in Real-World Patients With Acute Heart Failure Syndrome: A Prospective Nationwide Cohort Study
Source: Front Cardiovasc Med. 2022 Feb 22;9:791446. doi: 10.3389/fcvm.2022.791446 (PMC8902170; doi:10.3389/fcvm.2022.791446)
Supplement: Supplementary file 3 [file Table_3.DOCX]

**Supplementary material**

**The prescription characteristics, efficacy and safety of spironolactone in real-world patients with acute heart failure syndrome: A prospective nationwide cohort study**

Soo Jin Na, Jong-Chan Youn, Hye Sun Lee, Soyoung Jeon, Hae-Young Lee, Hyun-Jai Cho, Jin-Oh Choi, Eun-Seok Jeon, Sang Eun Lee, Min-Seok Kim, Jae-Joong Kim, Kyung-Kuk Hwang, Myeong-Chan Cho, Shung Chull Chae, Seok-Min Kang, Dong-Ju Choi, Byung-Su Yoo, Kye Hoon Kim, Byung-Hee Oh, Sang Hong Baek

**Table S3. Discontinuation of spironolactone at the first follow-up**

|  | Patients who prescribed spironolactone at discharge  (n = 1,324) |
| --- | --- |
| Discontinuation of spironolactone | 168 (12.7) |
| Cause of discontinuation |  |
| Hypotension | 10 (6.0) |
| Worsening heart failure | 4 (2.4) |
| Worsening renal function | 10 (6.0) |
| Hyperkalemia | 12 (7.1) |
| Gynecomastia | 0 (0.0) |
| Other | 39 (23.2) |
| Not recorded | 93 (55.4) |

Values are n (%).
