# Supplementary material for: The Prescription Characteristics, Efficacy and Safety of Spironolactone in Real-World Patients With Acute Heart Failure Syndrome: A Prospective Nationwide Cohort Study
Source: Front Cardiovasc Med. 2022 Feb 22;9:791446. doi: 10.3389/fcvm.2022.791446 (PMC8902170; doi:10.3389/fcvm.2022.791446)
Supplement: Supplementary file 4 [file Table_4.DOCX]

**Supplementary material**

**The prescription characteristics, efficacy and safety of spironolactone in real-world patients with acute heart failure syndrome: A prospective nationwide cohort study**

Soo Jin Na, Jong-Chan Youn, Hye Sun Lee, Soyoung Jeon, Hae-Young Lee, Hyun-Jai Cho, Jin-Oh Choi, Eun-Seok Jeon, Sang Eun Lee, Min-Seok Kim, Jae-Joong Kim, Kyung-Kuk Hwang, Myeong-Chan Cho, Shung Chull Chae, Seok-Min Kang, Dong-Ju Choi, Byung-Su Yoo, Kye Hoon Kim, Byung-Hee Oh, Sang Hong Baek

**Supplement Table S4. STEPP analysis**

| **Subpopulation** | **LVEF** | | |  | **No SPR** | | | **SPR** | | | **Difference** | | |
| --- | --- | --- | --- | --- | --- | --- | --- | --- | --- | --- | --- | --- | --- |
|  | Median | Minimum | Maximum | N | N | Survival probability | SE | N | Survival probability | SE | Survival probability | SE | p-value |
| 1 | 17.0 | 8.0 | 20.0 | 538 | 224 | 0.54 | 0.03 | 314 | 0.67 | 0.03 | -0.13 | 0.04 | 0.003 |
| 2 | 23.0 | 20.1 | 25.0 | 540 | 243 | 0.57 | 0.03 | 297 | 0.66 | 0.03 | -0.09 | 0.04 | 0.044 |
| 3 | 28.0 | 25.1 | 30.0 | 598 | 275 | 0.63 | 0.03 | 323 | 0.70 | 0.03 | -0.07 | 0.04 | 0.082 |
| 4 | 33.0 | 30.1 | 35.0 | 623 | 301 | 0.67 | 0.03 | 322 | 0.65 | 0.03 | 0.01 | 0.04 | 0.746 |
| 5 | 38.0 | 35.0 | 40.0 | 515 | 267 | 0.59 | 0.03 | 248 | 0.62 | 0.03 | -0.03 | 0.04 | 0.559 |
| 6 | 43.5 | 40.2 | 46.0 | 538 | 313 | 0.67 | 0.03 | 225 | 0.68 | 0.03 | -0.01 | 0.04 | 0.885 |
| 7 | 50.0 | 46.0 | 52.8 | 500 | 297 | 0.63 | 0.03 | 203 | 0.61 | 0.03 | 0.02 | 0.04 | 0.728 |
| 8 | 55.8 | 52.2 | 58.5 | 500 | 301 | 0.71 | 0.03 | 199 | 0.64 | 0.03 | 0.06 | 0.04 | 0.133 |
| 9 | 62.0 | 58.0 | 66.0 | 522 | 334 | 0.68 | 0.03 | 188 | 0.66 | 0.04 | 0.02 | 0.04 | 0.640 |
| 10 | 70.0 | 66.1 | 98.0 | 295 | 194 | 0.69 | 0.03 | 101 | 0.62 | 0.05 | 0.07 | 0.06 | 0.252 |
