# Supplementary material for: The Prescription Characteristics, Efficacy and Safety of Spironolactone in Real-World Patients With Acute Heart Failure Syndrome: A Prospective Nationwide Cohort Study
Source: Front Cardiovasc Med. 2022 Feb 22;9:791446. doi: 10.3389/fcvm.2022.791446 (PMC8902170; doi:10.3389/fcvm.2022.791446)
Supplement: Supplementary file 5 [file Table_5.DOCX]

**Supplementary material**

**The prescription characteristics, efficacy and safety of spironolactone in real-world patients with acute heart failure syndrome: A prospective nationwide cohort study**

Soo Jin Na, Jong-Chan Youn, Hye Sun Lee, Soyoung Jeon, Hae-Young Lee, Hyun-Jai Cho, Jin-Oh Choi, Eun-Seok Jeon, Sang Eun Lee, Min-Seok Kim, Jae-Joong Kim, Kyung-Kuk Hwang, Myeong-Chan Cho, Shung Chull Chae, Seok-Min Kang, Dong-Ju Choi, Byung-Su Yoo, Kye Hoon Kim, Byung-Hee Oh, Sang Hong Baek

**Supplement Table S5. Clinical and treatment characteristics in propensity-matched patients**

| **Variables** | **No SPR**  **(n=1,747)** | **SPR**  **(n=1,747)** | **P-value** |
| --- | --- | --- | --- |
| Age, years | 69.3±14.5 | 69.0±14.2 | 0.619 |
| Male | 882 (50.5) | 896 (51.3) | 0.636 |
| De novo HF | 943 (54.0) | 954 (54.6) | 0.709 |
| Past medical history |  |  |  |
| Hypertension | 1026 (58.7) | 1038 (59.4) | 0.680 |
| Diabetes mellitus | 586 (33.5) | 599 (34.3) | 0.642 |
| Ischemic heart disease | 498 (28.5) | 462 (26.5) | 0.179 |
| Dilated cardiomyopathy | 115 (6.6) | 152 (8.7) | 0.019 |
| Valvular heart disease | 246 (14.1) | 227 (13.0) | 0.351 |
| Atrial fibrillation | 479 (27.4) | 502 (28.7) | 0.387 |
| Chronic lung disease | 184 (10.5) | 200 (11.5) | 0.384 |
| Chronic renal failure | 306 (17.5) | 139 (8.0) | <0.001 |
| Cerebrovascular disease | 261 (14.9) | 254 (14.5) | 0.738 |
| Treatment during hospitalization |  |  |  |
| Parenteral diuretics | 1318 (75.4) | 1417 (81.1) | <0.001 |
| Parenteral inotropes | 470 (26.9) | 482 (27.6) | 0.648 |
| Parenteral vasodilators | 794 (45.4) | 733 (42.0) | 0.038 |
| Intensive care unit admission | 839 (48.0) | 806 (46.1) | 0.263 |
| Mechanical ventilation | 227 (13.0) | 209 (12.0) | 0.357 |
| Renal replacement therapy | 134 (7.7) | 42 (2.4) | <0.001 |
| Vital signs at discharge |  |  |  |
| Systolic blood pressure, mmHg | 114.4±16.6 | 114.4±17.0 | 0.997 |
| Heart rate, /min | 76.5±14.2 | 76.5±14.2 | 0.939 |
| NYHA class II-IV | 1363 (80.7) | 1403 (82.2) | 0.279 |
| Laboratory measurements at discharge |  |  |  |
| Sodium, mmol/L | 137.8±3.9 | 137.8±3.9 | 0.908 |
| Potassium, mmol/L | 4.2±0.5 | 4.2±0.5 | 0.001 |
| Hemoglobin, g/dL | 11.8±2.1 | 12.3±2.1 | <0.001 |
| Creatinine, mg/dL | 1.5±1.5 | 1.1±0.7 | <0.001 |
| Ejection fraction, % | 41.8±16.2 | 39.0±15.1 | <0.001 |
| CRP > 3mg/dL or hs-CRP > 10mg/dL | 199 (11.4) | 208 (11.9) | 0.635 |
| BNP > 100pg/mL or NT-proBNP > 360pg/mL | 1672 (95.7) | 1674 (95.8) | 0.867 |

Values are mean ± standard deviation and median with interquartile range or n (%).

BNP indicates brain natriuretic peptide; CRP, C-reactive protein; HF, heart failure; hs-CRP, high-sensitivity C-reactive protein; NT-proBNP, N-terminal pro-brain natriuretic peptide; NYHA, New York Heart Association; SPR, spironolactone.
