# Supplementary figures and images for: The Prescription Characteristics, Efficacy and Safety of Spironolactone in Real-World Patients With Acute Heart Failure Syndrome: A Prospective Nationwide Cohort Study
Source: Front Cardiovasc Med. 2022 Feb 22;9:791446. doi: 10.3389/fcvm.2022.791446 (PMC8902170; doi:10.3389/fcvm.2022.791446)

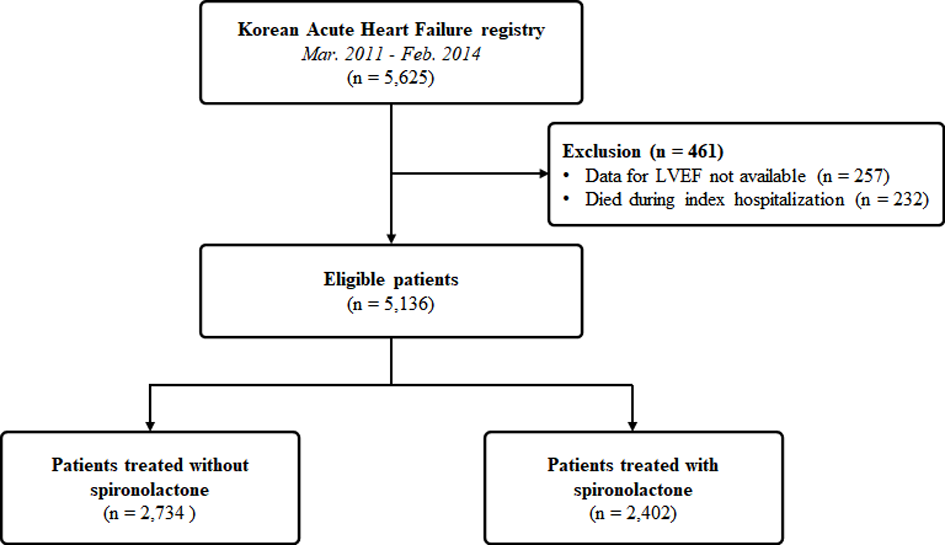

Supplement: Supplementary file 6 [file Image_1.TIF]

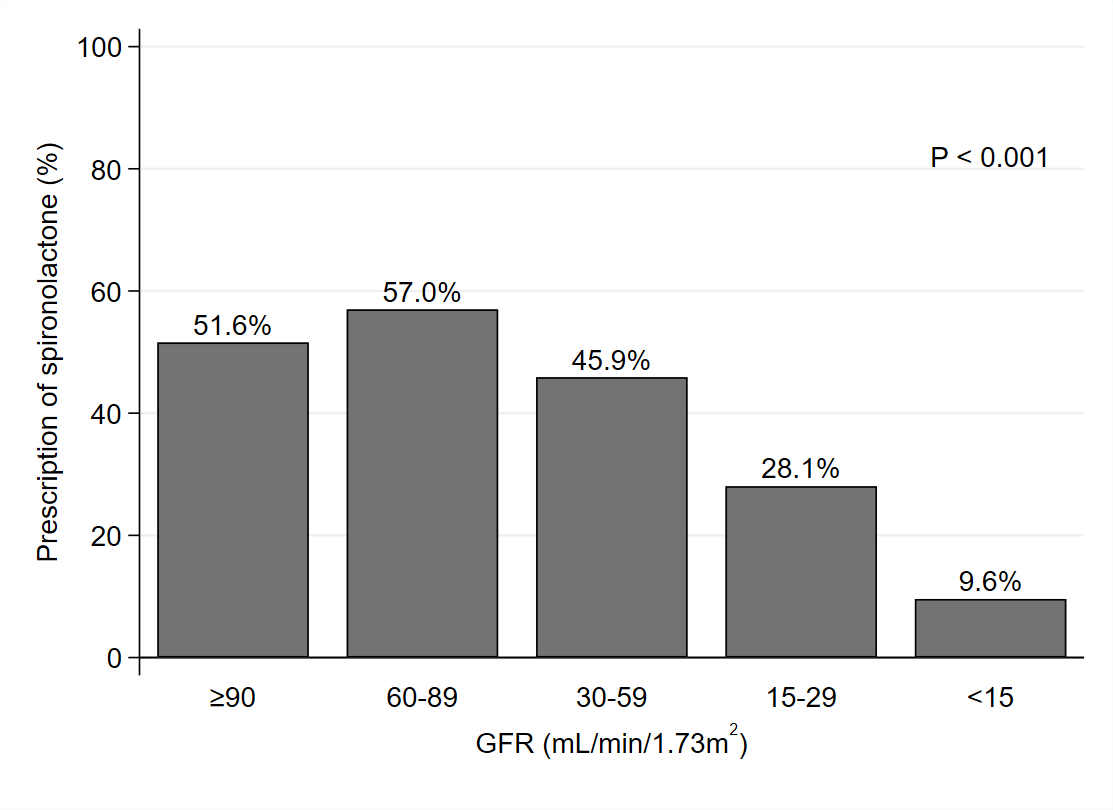

Supplement: Supplementary file 7 [file Image_2.TIF]

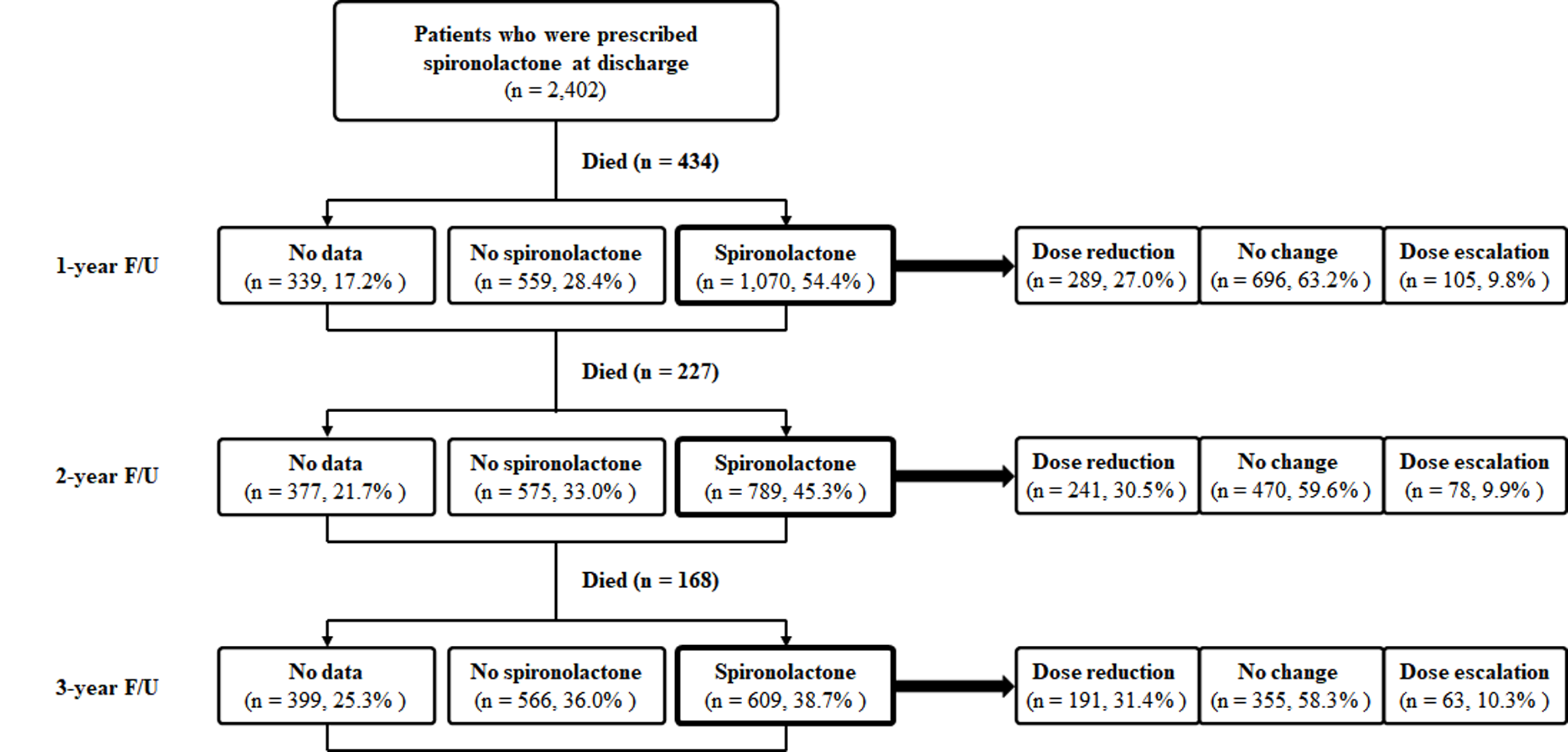

Supplement: Supplementary file 8 [file Image_3.TIF]

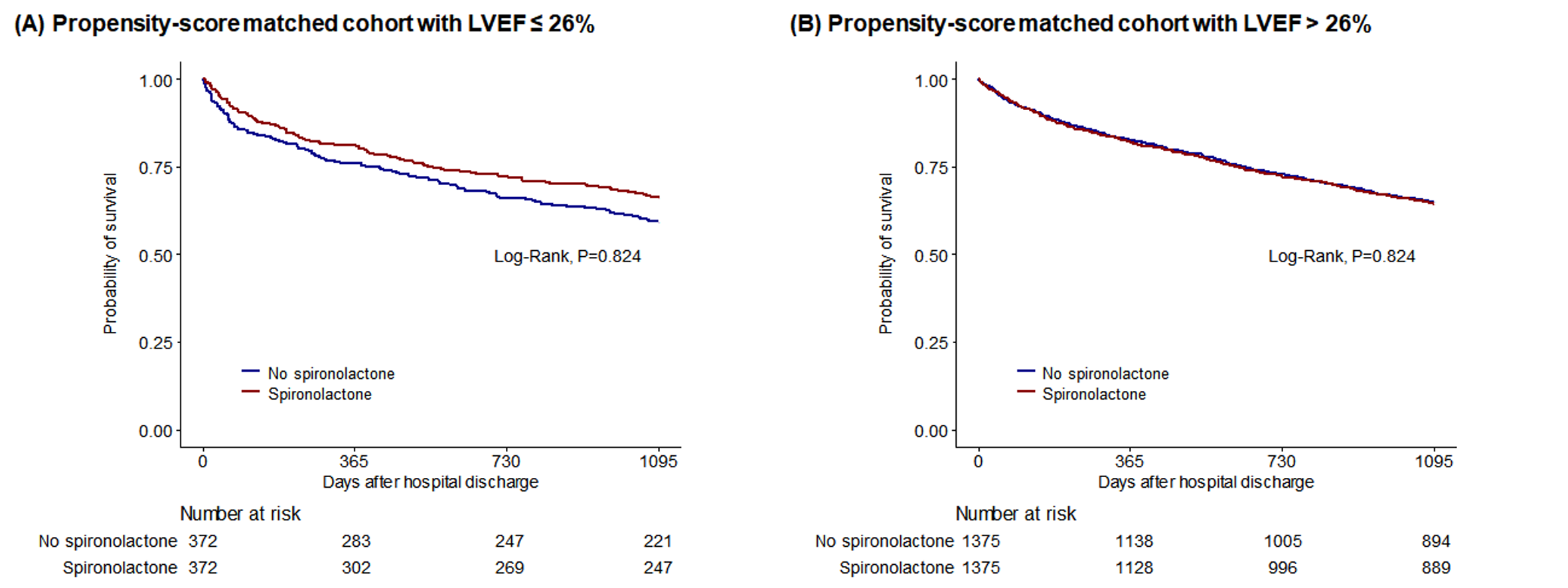

Supplement: Supplementary file 9 [file Image_4.TIF]
